# The Interfacial Characteristics of Graphene/Al_4_C_3_ in Graphene/AlSi10Mg Composites Prepared by Selective Laser Melting: First Principles and Experimental Results

**DOI:** 10.3390/ma13030702

**Published:** 2020-02-04

**Authors:** Wenjie Zhao, Zhanyong Zhao, Peikang Bai, Lizheng Zhang, Bing Han, Wenbo Du

**Affiliations:** 1 School of Materials Science and Engineering, North University of China, Taiyuan 030051, China; nuczhaowenjie@163.com (W.Z.); 18435841275@163.com (L.Z.); 2 College of Mechatronics Engineering, North University of China, Taiyuan 030051, China; chihb2008@live.cn; 3 National Key Laboratory for Remanufacturing, Academy of Army Armored Forces, Beijing 100072, China; dwbneu@163.com

**Keywords:** selective laser melting, graphene, first principle, interfacial bonding, interface alloying

## Abstract

The Al_4_C_3_ phase was precipitated via a reaction of graphene (Gr) with Al during selective laser melting (SLM). The interfacial nature of the Gr (0001)/Al_4_C_3_ (0001) interface was determined using the first-principle calculation. The simulation results showed that the influence of the stacking site on the interfacial structure was limited and the Al-termination interface presented a more stable structure than the C-termination interface. The Al-termination-CH site interface had the largest work of adhesion (6.28 J/m^2^) and the smallest interfacial distance (2.02 Å) among the four interfacial structures. Mulliken bond population analysis showed that the bonding of the Al-termination interface was a mixture of covalent and ionic bonds and there was no chemical bonding in the C-termination interface.

## 1. Introduction

The excellent mechanical, electrical, and optical properties of graphene (Gr) make it a new material with wide applications [1,2]. With its the increasing application in various fields, graphene has also attracted a lot of attention in relation to the performance of modified Al composites [3,4]. There are many ways to prepare graphene/Al, such as powder metallurgy, liquid stir casting, pressure infiltration, accumulative roll bonding, and friction stir processing. Li et al., prepared graphene nano-platelets/Al composites using the powder metallurgy technique [5], whereas Huang et al., prepared graphene-reinforced Al-based nanocomposites with excellent hardness and tensile strength by employing the high-pressure torsion method [6]. Moreover, Shao et al., prepared 5083 Al matrix composites reinforced with graphene oxide and graphene nanoplates via the pressure infiltration method [7]. However, these methods are limited when preparing complex parts. As an advanced manufacturing method, selective laser melting (SLM) technology has great advantages in manufacturing complex parts because of its high precision and low cost. Hu et al., prepared graphene/aluminum nanocomposites and found that the hardness of the composites was greatly enhanced [8]. We have also prepared high-performance Gr/Al composites through the SLM process [9,10].

The first-principle calculation method can simulate materials at the atomic scale and has been widely used to study the properties of materials [11,12]. By using first-principle calculations, Li et al., investigated the heterogeneous nucleation interface of Al/Al_3_Ti [13] and Wang et al., examined the interfacial properties of the Mg (0002)/Al_2_MgC_2_ (0001) interface [14]. It has also been shown that Al_4_C_3_ might nucleate and grow on graphene, although this needs to be further confirmed [15,16]. The interaction of atoms at the interface between Gr and Al_4_C_3_ has not been studied, as far as we know. Based on metal solidification and the thermodynamic theory, a relatively stable nucleation interface needs a larger work of adhesion and a smaller interfacial energy, which will directly affect the potency of a heterogeneous substrate [17]. Therefore, a deeper investigation on Gr/Al_4_C_3_ interfacial structures at the atomic scale is necessary.

In this research, Gr (0001) and Al_4_C_3_ (0001) were studied because of their regular hexagonal atomic arrangement and relative small surface energy [18,19]. The main purpose of this paper was to analyze Gr/Al_4_C_3_ surface and interfaces by carrying out first-principle calculations and to discuss the potential of graphene as a heterogeneous nucleation substrate for Al_4_C_3_, on the basis of the calculation results. Our study could be highly significative for the interpretation of experimental results regarding graphene-reinforced Al-based composites and provide theoretical guidance for subsequent experiments.

## 2. Computational and Experimental Procedure

The graphene used in the experiment was provided by Renishaw Plc. (Renishaw, UK). AlSi10Mg powders were supplied by Tangshan Jianhua Science and Technology Development Co. Ltd.,(Tangshan, China). More information about the experimental materials can be found in our previous work [9]. The Gr/AlSi10Mg composites were produced by the Renishaw AM400 (Renishaw, England). The composites were produced with a laser power of 300W, a scanning speed of 1200 mm/s, a hatch spacing of 130 μm, and a layer thickness of 30 μm. The microstructures of the composites were analyzed using a scanning electron microscope (SEM, Zeiss Ultra 55, Jena, Germany), which was equipped with an energy dispersive spectroscope (EDS). After a thinning treatment, the microstructure of the composites was observed using high-resolution transmission electron microscopy (HRTEM, JEM-F200, Tokyo, Japan).

**Figure 1:**
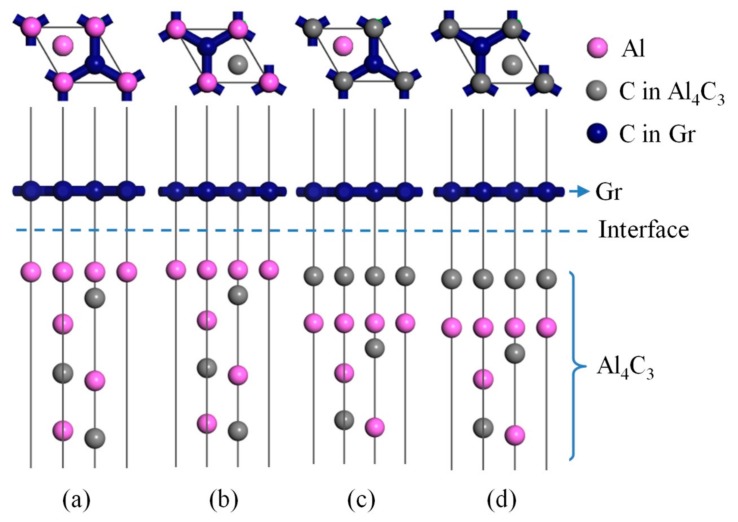
Crystal structures of graphene (Gr) (0001)/Al_4_C_3_ (0001) interfaces. (**a**) Al-termination-AH-site, (**b**) Al-termination-CH-site, (**c**) C-termination-AH-site, (**d**) C-termination-CH-site.

The first principle based on the density functional theory was employed in this experiment. The simulation was based on the Cambridge Sequential Total Energy Package (CASTEP) code, which employed ultrasoft pseudopotentials to represent the interactions between valence electrons and the ionic core [20]. The atoms were relaxed to obtain the minimum energy of the system by solving the Broyden Fletcher Goldfarb Shanno (BFGS) function [21]. According to our previous work, a 10-layer Al-termination slab and a 12-layer C-termination Al_4_C_3_ (0001) slab were placed on a single-layer Gr (0001) slab [22]; in addition, a 10 Å vacuum was placed on the top of the Gr (0001) slab to prevent the periodic influence of free surfaces [23]. A model showing the interface between Gr and Al_4_C_3_ is presented in Figure 1.

## 3. Results and Discussion

**Figure 2:**
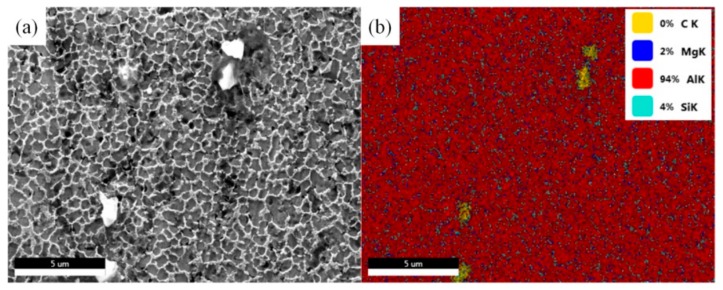
(**a**) SEM images of Gr/AlSi10Mg composites, (**b**) EDS mapping of the C, Mg, Al, and Si elements in (**a**).

**Figure 3:**
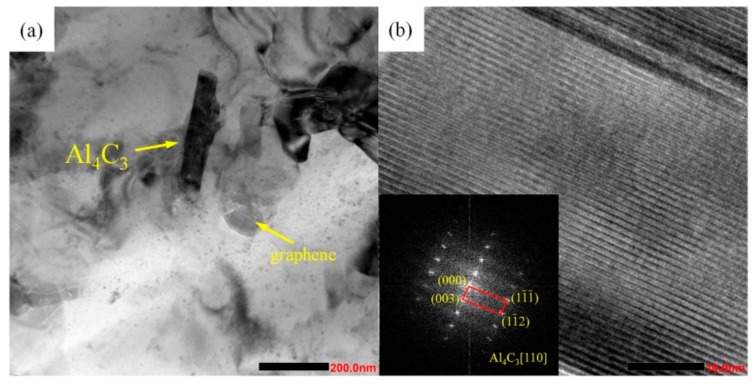
TEM images of Gr/AlSi10Mg composites. (**a**) Low-magnification image, (**b**) HRTEM observation of Al_4_C_3_; the insets show the FFT (Fast Fourier transform) patterns of Al_4_C_3_.

As shown in Figure 2 (a), the microstructure of Gr/AlSi10Mg composites formed by SLM showed a typical cellular eutectic morphology caused by fast solidification [24]. Figure 2b shows the EDS mapping of the C, Mg, Al, and Si elements of Figure 2a. The TEM images of Gr/AlSi10Mg are shown in Figure 3. In Figure 3a, the Al_4_C_3_ phase, which had been verified by the FFT (Fast Fourier transform) pattern in Figure 3b, was observed near the interface between Gr and the Al matrix, as reported in several articles [5,15,25].

In this experiment, high-quality monolayer graphene was used to synthesize the graphene-reinforced AlSi10Mg composite. For this, monolayer graphene was selected to build the interfacial model. The surface energy of graphene can be expressed as [17,26,27]:

(1) $$E_{\mathrm{surf}}=\frac{1}{2A}\left(E_{\mathrm{slab}}\left(N\right)-{\mathrm{NE}}_{\mathrm{bulk}}\right)$$

where E_slab_ (N) is the total surface energy, A is the surface area, N is the number of atoms in the surface slab, and E_bulk_ is the energy per atom in the bulk. The surface energy of the Gr (0001) slab was 0.012 J/m^2^. The surface energy of Al_4_C_3_ can be expressed by [18,28]:

(2) $$E_{{\mathrm{Al}}_{4}C_{3}}=\frac{1}{2A}\left[E_{\mathrm{slab}}-N_{\mathrm{Al}}\mu_{\mathrm{Al}}-N_{C}\mu_{C}+\mathrm{PV}-\mathrm{TS}\right]$$

where E_slab_ is the total energy of the fully relaxed Al_4_C_3_ (0001) surface slab, A is the surface area, and μ_Al_ and μ_C_ are the chemical potential of the aluminum atom and carbon atom in the surface slab, respectively. N_Al_ and N_C_ are the numbers of the corresponding atoms in the surface slab. According to our previous work, the surface energy of Al_4_C_3_ changed from 1.64 to 1.47 J/m^2^ for Al-termination and from 5.73 to 6.23 J/m^2^ for C-termination [22].

The work of adhesion for Gr (0001)/Al_4_C_3_ (0001) was calculated using the formula [29,30]:

(3) $$W_{\mathrm{ad}}=\frac{1}{A}\left(E_{\mathrm{total}}^{\mathrm{Gr}}+E_{\mathrm{total}}^{{\mathrm{Al}}_{4}C_{3}}-E_{\mathrm{total}}^{\mathrm{Gr}/{\mathrm{Al}}_{4}C_{3}}\right)$$

where $E_{\mathrm{total}}^{\mathrm{Gr}}$ and $E_{\mathrm{total}}^{{\mathrm{Al}}_{4}C_{3}}$ are the total energy of the fully relaxed surface slabs, $E_{\mathrm{total}}^{\mathrm{Gr}/{\mathrm{Al}}_{4}C_{3}}$ is the total energy of the Gr/Al_4_C_3_ interface, and A is the surface area.

The work of adhesion and interfacial distance of four Gr (0001)/Al_4_C_3_ (0001) interfaces before and after relaxation are reported in Table 1. The interfacial distance of the Al-termination interface after relaxation was smaller than the initial distance, while the interfacial distance of the C-termination interface increased. This shows that the type of termination has a great impact on the interface [31]. Stacking sites had a little impact on the interface. The Al-termination interface had a larger work of adhesion than the C-termination interface, while the Al-termination-CH-site interface had the largest work of adhesion (6.28 J/m^2^) and the smallest interfacial distance (2.02 Å).

The interfacial energy of the Gr (0001)/Al_4_C_3_ (0001) interface can be defined as [32]:

(4) $$\gamma =\frac{1}{A}\left[E_{\mathrm{total}}+\left(\frac{4}{3}N_{C,1}-N_{\mathrm{Al}}\right)\mu_{\mathrm{Al}}-\frac{1}{3}N_{C,1}\mu_{{\mathrm{Al}}_{4}C_{3}}^{\mathrm{bulk}}-N_{C,2}\mu_{\mathrm{Gr}}^{\mathrm{bulk}}\right]-\delta_{\mathrm{Gr}}-\delta_{{\mathrm{Al}}_{4}C_{3}}$$

where E_total_ is the total energy of the interfacial structure, and N_C,1_ and N_C,2_ are the number of carbon atoms in the Al_4_C_3_ and Gr surface models, respectively. N_Al_ is the number of aluminum atoms in the surface model of Al_4_C_3_, $\mu_{{\mathrm{Al}}_{4}C_{3}}^{\mathrm{bulk}}$ and $\mu_{\mathrm{Gr}}^{\mathrm{bulk}}$ are the chemical potentials of the bulk Al_4_C_3_ and Gr, and $\delta_{\mathrm{Gr}}$ and $\delta_{{\mathrm{Al}}_{4}C_{3}}$ are the surface energies of the Gr and Al_4_C_3_ surface structures.

**Figure 4:**
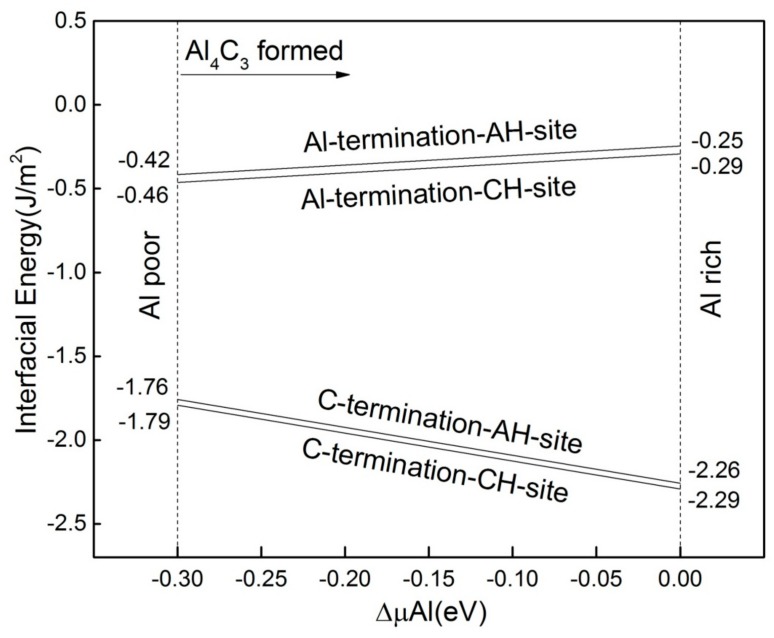
Interfacial energies of four interfacial structures as a function of ΔμAl.

The results showed that the maximum and the minimum of the interfacial energy were −0.25 J/m^2^ and −2.29 J/m^2^, respectively (Figure 4). Interfaces with negative interface energy are not stable in thermodynamics. When the negative value of interfacial energy is high enough, it can provide a driving force promoting the diffusion through the interface of the atoms positioned near it. This will result in interfacial alloying and in the formation of a new interfacial phase [33]. Therefore, the C-termination interface has a higher tendency to further react and form a stable interface.

**Figure 5:**
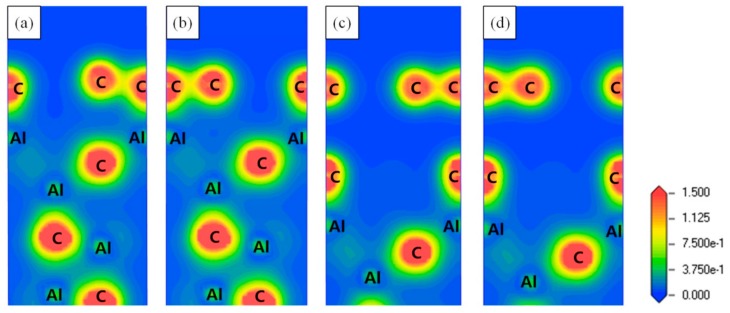
Charge density for the four Gr (0001)/Al_4_C_3_ (0001) interfaces taken along the ($11\bar{2}0$) direction. (**a**) Al-termination-AH-site interface, (**b**) Al-termination-CH-site interface, (**c**) C-termination-AH-site interface, (**d**) C-termination-CH-site interface.

**Figure 6:**
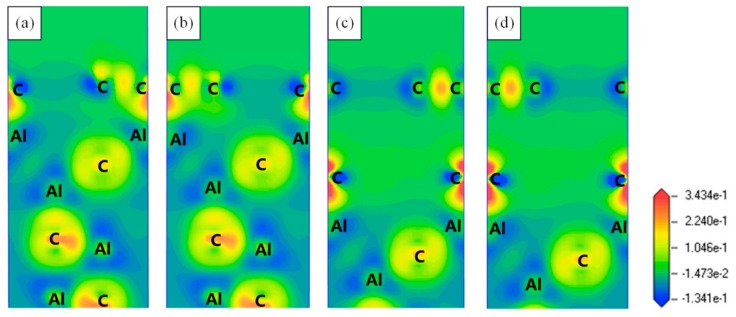
Charge density difference for the four Gr (0001)/Al_4_C_3_ (0001) interfaces taken along the ($11\bar{2}0$) direction. (**a**) Al-termination-AH-site interface, (**b**) Al-termination-CH-site interface, (**c**) C-termination-AH-site interface, (**d**) C-termination-CH-site interface.

In order to investigate the interfacial bonding nature of the Gr (0001)/Al_4_C_3_ (0001) interfaces, the charge density distributions and the charge density differences for the four Gr (0001)/Al_4_C_3_ (0001) interfaces were examined, as shown in Figure 5 and Figure 6, respectively. The results showed that the effect of the stacking site was limited, which led to no difference in charge distribution between the AH-site and the CH-site in the same termination interface. For the Al-termination interface, a wide range of charge accumulation regions existed in the interfacial Al atom. The lost charge was transferred to the interfacial C atom of the Gr side, proving certain ionic features of the Al-termination interface. Because of the large the distance between the graphene layer and the Al_4_C_3_ layer in the C-termination interface, there was no obvious regionalization feature at the interface.

To further clarify the interfacial bonding characteristics of the Gr (0001)/Al_4_C_3_ (0001) interfaces, the Mulliken population was analyzed. Table 2 lists the Mulliken population analysis results of the Al and C atoms both at the interface and at the free surface. For the Al-termination interfaces, the interfacial Al atom lost more charge compared with the Al atom of the Al-termination free surface, while the interfacial C atom gained more charge. This indicates that an ionic bonding existed between the interfacial Al atom and the C atom. The overlap populations of the Al-C bond in the two Al-termination interfaces were of 0.31 and 0.19 respectively, which proves that covalent bonding was formed at the interface. Therefore, the bonding of the Al-termination interfaces was a mixture of covalent bonds and ionic bonds. For the C-termination interfaces, although there were gains and losses of charge in the interfacial C atoms, the Mulliken bond population analysis results showed that there was no chemical bonding at the interface.

## 4. Conclusions

The Al_4_C_3_ phase was precipitated near graphene due to the reaction of graphene with Al during the SLM process. The first-principle calculation results showed that the Al-termination interface had larger work of adhesion and smaller interfacial energy and presented a mixture of covalent and ionic bonds at the Gr (0001)/Al_4_C_3_ (0001) interface. The work of adhesion of the C-termination interface was smaller, and there was no chemical bond at the interface, while the atoms near the C-termination interfaces were more likely to diffuse through the interface to produce interface alloying, which had an extremely important role in improving the stability of the Gr (0001)/Al_4_C_3_ (0001) interface. Based on the above experimental and first-principle calculations results, it can be concluded that graphene can be an effective nucleation substrate for Al_4_C_3_. This study will provide a theoretical reference for future research of Gr/Al composites.

## Figures and Tables

**Table 1:** Work of adhesion and interfacial distance for the unrelaxed and relaxed Gr (0001)/ Al_4_C_3_ (0001) interfaces.

Termination	Stacking	Unrelaxed		Relaxed	
		d_0_ (Å)	W_ad_ (J/m^2^)	d_1_ (Å)	W_ad_ (J/m^2^)
Al	AH	3.11	−0.36	2.05	5.98
	CH	3.11	−0.45	2.02	6.28
C	AH	3.40	0.29	3.48	0.26
	CH	3.40	0.28	3.49	0.79

**Table 2:** Mulliken population analysis results of the nearest-neighbor atoms at the interface and atoms at the free surface (eV).

System	Atom	s	p	Total	Charge
Al-termination-AH interface	Al	0.71	1.16	1.87	+1.13
	C	1.42	2.88	4.30	−0.30
Al-termination-CH interface	Al	0.66	1.15	1.82	+1.18
	C	1.43	2.87	4.30	−0.30
C-termination-AH interface	C ^a^	1.87	2.51	4.38	−0.38
	C ^b^	1.35	2.63	3.97	+0.03
C-termination-CH interface	C ^a^	1.88	2.50	4.38	−0.38
	C ^b^	1.34	2.63	3.97	+0.03
Al-termination free surface	Al	1.06	1.22	2.29	+0.71
C-termination free surface	C	1.87	2.51	4.38	−0.38
Gr free surface	C	1.05	2.95	4.00	0

^a^ C atom from the Al_4_C_3_ side. ^b^ C atom from the Gr side.
